# Caspase-dependent apoptosis in Riboflavin Transporter Deficiency iPSCs and derived motor neurons

**DOI:** 10.1038/s41420-024-01812-y

**Published:** 2024-01-26

**Authors:** Chiara Marioli, Maurizio Muzzi, Fiorella Colasuonno, Cristian Fiorucci, Nicolò Cicolani, Stefania Petrini, Enrico Bertini, Marco Tartaglia, Claudia Compagnucci, Sandra Moreno

**Affiliations:** 1 Department of Science, LIME, University Roma Tre, 00146 Rome, Italy; 2 Molecular Genetics and Functional Genomics, Ospedale Pediatrico Bambino Gesù, IRCCS, 00146 Rome, Italy; 3 Laboratory of Neurodevelopment, Neurogenetics and Neuromolecular Biology, IRCCS Santa Lucia Foundation, 00179 Rome, Italy; 4 Department of Experimental Medicine, University of Rome “Tor Vergata”, 00133 Rome, Italy; 5 Confocal Microscopy Core Facility, Research Laboratories, IRCCS Ospedale Pediatrico Bambino Gesù, 00146 Rome, Italy; 6 Unit of Neuromuscular and Neurodegenerative Disorders, IRCCS Ospedale Pediatrico Bambino Gesù, 00146 Rome, Italy

**Keywords:** Cellular neuroscience, Induced pluripotent stem cells, Motor neuron disease, Cell death in the nervous system

## Abstract

Riboflavin Transporter Deficiency (RTD) is a rare genetic, childhood-onset disease. This pathology has a relevant neurological involvement, being characterized by motor symptoms, ponto-bulbar paralysis and sensorineural deafness. Such clinical presentation is associated with muscle weakness and motor neuron (MN) degeneration, so that RTD is considered part of the MN disease spectrum. Based on previous findings demonstrating energy dysmetabolism and mitochondrial impairment in RTD induced Pluripotent Stem cells (iPSCs) and iPSC-derived MNs, here we address the involvement of intrinsic apoptotic pathways in disease pathogenesis using these patient-specific in vitro models by combined ultrastructural and confocal analyses. We show impaired neuronal survival of RTD iPSCs and MNs. Focused Ion Beam/Scanning Electron Microscopy (FIB/SEM) documents severe alterations in patients’ cells, including deranged mitochondrial ultrastructure, and altered plasma membrane and nuclear organization. Occurrence of aberrantly activated apoptosis is confirmed by immunofluorescence and TUNEL assays. Overall, our work provides evidence of a role played by mitochondrial dysfunction in RTD, and identifies neuronal apoptosis as a contributing event in disease pathogenesis, indicating intrinsic apoptosis pathways as possible relevant targets for more effective therapeutical approaches.

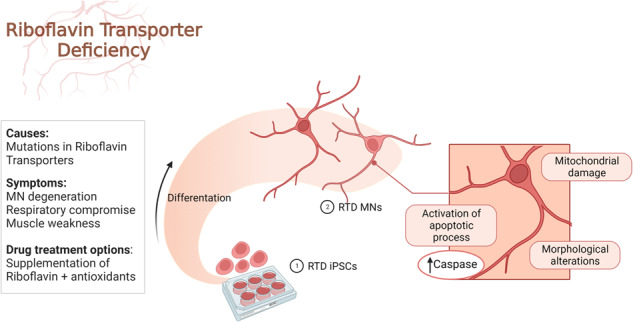

## Introduction

Riboflavin Transporter Deficiency (RTD) is an early onset rare motor neuron (MN) disease caused by variants in *SLC52A3* and *SLC52A2* genes, encoding the RFVT3 and RFVT2 riboflavin (RF) transporters, respectively [1]. Though clinical variability of the disorder has been reported, RTD patients generally suffer from neuropathy, hearing loss, bulbar palsy and respiratory compromise, whereas their cognitive functions are invariably preserved [2]. Mutations causing RTD include frameshift, missense, nonsense, and splice-site alterations, but uniformly result in reduced RF uptake and systemic distribution. Indeed, RF is a water-soluble group B vitamin that cannot be endogenously synthesized by mammalian cells, thus needing to be absorbed in the gastrointestinal tract by RFVT3 and transported into the bloodstream and to target tissues by RFVT1, 2 and 3 [3, 4]. RF derivatives, flavin adenine dinucleotide (FAD) and flavin mononucleotide (FMN), play a role in vital biological reactions, including carbohydrate, lipid and amino acid metabolism, many of which involving mitochondria [5, 6].

RTD is characterized by specific degeneration of MNs. Induced pluripotent stem cells (iPSCs) and iPSC-derived MNs have demonstrated to represent invaluable models to investigate the cellular pathophysiology of RTD [7]. By using these in vitro experimental systems, we previously demonstrated occurrence of altered energy metabolism pathways linked to dysregulated mitochondrial and peroxisome function, and defective cytoskeletal arrangement [8–11]. Notably, we also observed an indirect evidence of aberrant programmed cell death [9]. Such process is very likely to occur in RTD, in analogy to other neurodegenerative diseases [12]. Apoptosis plays a major physiological role in nervous tissue homeostasis, neurodevelopment and neurodegeneration [13]. In central nervous system (CNS) development, neurogenesis is accompanied by massive neuronal loss, mainly occurring during synaptogenesis [14]. Besides, in mature CNS, extensive neuronal cell death only occurs when homeostasis is lost, leading to inappropriate triggering of apoptotic pathways [12]. In this case, several factors, such as oxidative stress, mitochondrial dysfunction, alteration of Ca^2+^ homeostasis and lack of neurotrophic factors can determine the gradual loss of specific subsets of neurons and contribute to neurodegenerative disorders such as Alzheimer’s disease, Parkinson’s disease, amyotrophic lateral sclerosis, and spinal muscular atrophy [15–17].

Typically, apoptotic signals lead to the activation of a family of cysteine-aspartate proteases, known as caspases. While the extrinsic pathway is activated by binding of extracellular ligands to death receptors, the intrinsic pathway is initiated by internal stimuli, such as DNA damage and upregulation of pro-apoptotic factors [18]. Whatever the trigger, processes converge in cleavage and consequent activation of effector caspase 3, in turn activating substrates, such as DNases, responsible for nuclear DNA fragmentation [19]. In the nervous tissue, mitochondrial-driven apoptosis is a major pathway occurring in either physiological or pathological conditions. Upon Mitochondrial Outer Membrane Permeabilization (MOMP), crucial event of apoptotic pathways, Cyt *C* is released from the intermembrane space leading to apoptosome formation, which initiates cell breakdown. Other caspase-independent cell death pathways have also been described [20]. One of them involves apoptosis-inducing factor (AIF), a flavoprotein oxidoreductase, normally regulating electron transport chain assembly and stability, which is released upon MOMP and translocates to the nucleus where it participates in apoptotic chromatin lysis [21, 22].

Besides DNA fragmentation, apoptosis is characterized by morphological features which can be detected by light and electron microscopy. These include cell shrinkage and progressive pyknosis, due to chromatin marginalization and compacting. During this process, blebs, containing organelles and other cell components, protrude and detach from the plasma membrane forming vesicles, known as apoptotic bodies, readily visible under scanning electron microscope. These are rapidly phagocytosed by macrophages or surrounding cells [23].

Based on these considerations and previous findings, we investigated the occurrence of aberrantly activated cell death mechanisms by combined ultrastructural and confocal analyses approaches, using patient-specific iPSC and derived MNs as model systems.

## Results

### iPSCs

**Fig. 1:**
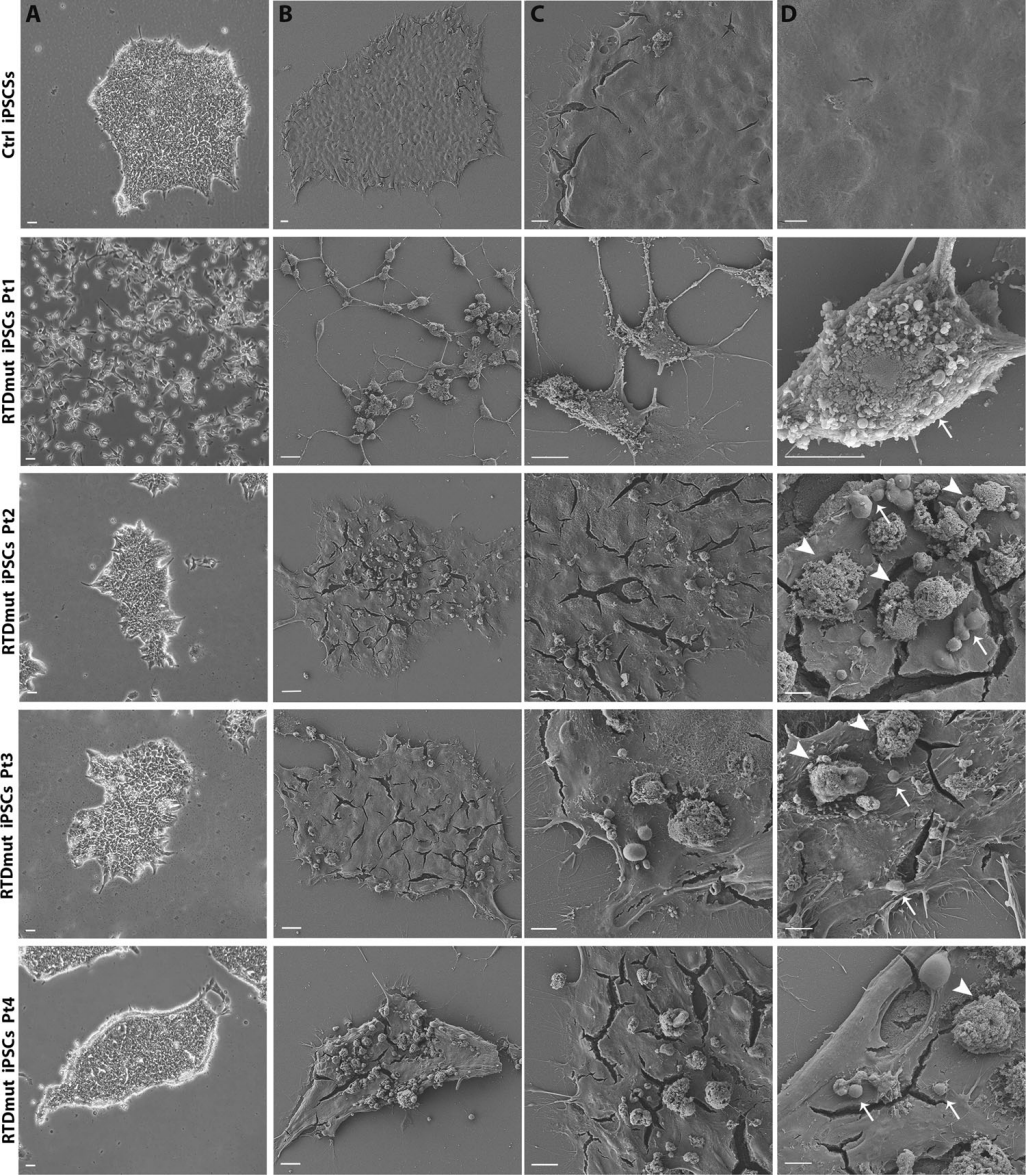
SEM micrographs of RTD iPSCs, captured using a TDL detector. The surface of Ctrl iPSCs appears smooth, unlike RTD patients’ cells, which show numerous blebs and vesicles at the apical surface. The right column displays higher magnification images, with blebs and vesicles indicated by white arrows and arrowheads, respectively. Scale bar **A** column, 20 µm; **B** column, 20 µm; **C** column, 10 µm; **D** column, 5 µm.

Morphological examination of cell cultures revealed remarkable differences between control (Ctrl) and RTD iPSCs. Phase contrast microscopic analysis demonstrates reduced ability to form colonies shown by RTD iPSCs, compared to healthy cell cultures, which instead showed regular margins of the colonies and stable cell-cell contacts as expected [9]. Notwithstanding general common features, RTD cells displayed patient-specific morphological abnormalities, in that RTDmut Pt1 iPSCs were totally disaggregated, while RTDmut Pt2, Pt3 and Pt4 form small, irregular colonies (Fig. 1).

These alterations, related to altered cell morphology, suggest cell suffering and/or ongoing death process. Based on these observations and previous findings [9–11] suggesting impaired survival of RTD cells, we addressed the putative involvement of apoptotic process in RTD patients’ iPSCs. Morphological details of RTD iPSCs were investigated by scanning electron microscopy (SEM), revealing abundant membrane blebbings in RTD iPSCs; on the contrary, Ctrl iPSCs showed smooth surfaces (Fig. 1). Additionally, spherical vesicles, ranging from 1 to 5 μm, which are reminiscent of apoptotic bodies, were consistently found to accumulate on apical cell surface in all RTD patients’ cell cultures.

**Fig. 2:**
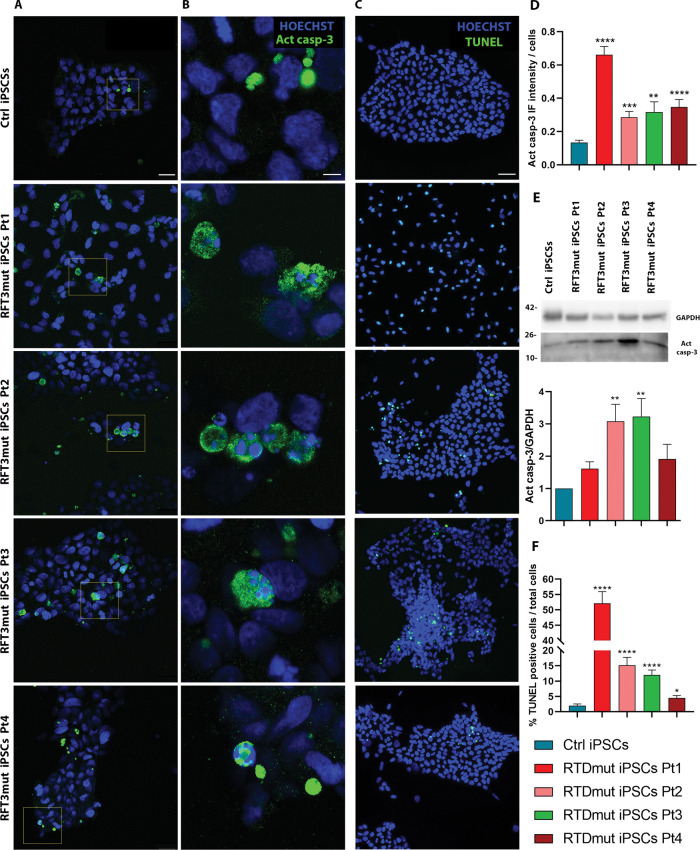
Analysis of apoptotic process in RTD iPSCs. Act casp-3 IF (Scale bar in **A**, 25 µM; in **B**, 5 µM). **C** TUNEL assay (Scale bar, 50 µm). Nuclei are stained with HOECHST (blue) while TUNEL and Act casp-3 positive cells are marked in green. **D** Quantification of Act casp-3 fluorescent signal intensity. A minimum of *n* = 3 and 9 images were quantized. **E** WB analysis of Act casp-3 and GAPDH (as loading control). Bar graph represents the mean ± SEM of three experiments (*n* = 3). **F** Quantitative analysis of TUNEL apoptotic assay (performed in triplicate). A minimum of 12 images and 4000 cells were counted. Data were analyzed by Student’s t tests and Kruskal-Wallis test ± SEM. ****p ≤ 0.0001; ***p ≤ 0.001; **p ≤ 0.01; *p ≤ 0.05.

We were then prompted to investigate the apoptotic pathway, particularly caspase activation, studying Caspase 3 immunoreactivity in RTD iPSCs, as compared to controls. Figure 2A, B displays representative confocal images of Activated Caspase 3 (Act casp-3) immunofluorescent localization, providing evidence of an abnormally high number of positive cells in RTD cultures. We also observed a heterogeneous intracellular distribution of the apoptotic marker, which was found in the peripheral cytoplasm, next to the plasma membrane. Notably, Act casp-3 positive cells often displayed morphological alterations typical of the apoptotic process, including blebs, apoptotic bodies, and nuclear aberration/fragmentation (Fig. 2B). When quantitatively evaluated, Act casp-3 signal intensity proved significantly higher in iPSCs of all RTD patients, as compared with Ctrl cells. However, individual-based differences in the abundance of Act casp-3 were also detected. Indeed, Pt1 cells display the highest fluorescence intensity levels, while Pt3 cells were the least immunoreactive (Fig. 2D). These data were consistent with WB findings (Fig. 2E). We performed TUNEL assay to evaluate apoptotic cell death. Confocal microscopic images showed the presence of a greater level of the nuclear fluorescent signal in RTD iPSCs, compared to Ctrl cells (Fig. 2C, F).

### iPSC-derived MNs

**Fig. 3:**
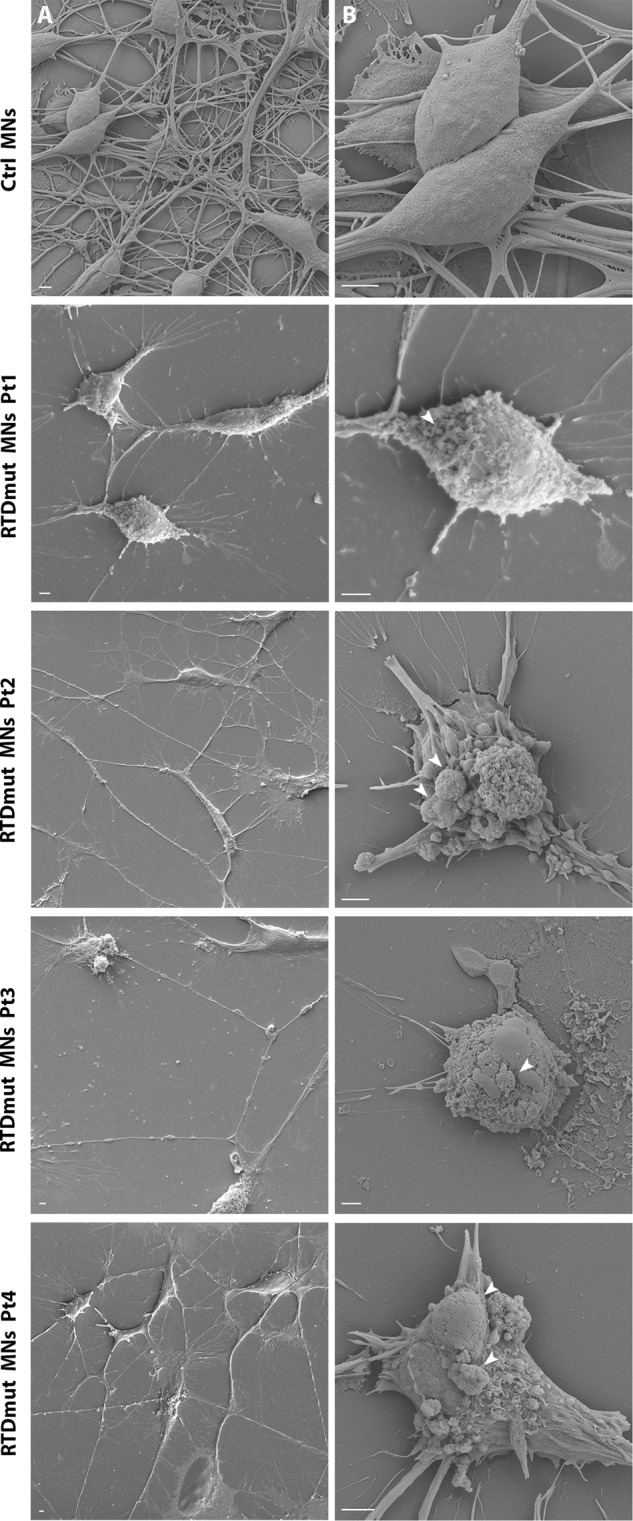
SEM micrographs of RTD MNs. Apoptotic blebs and bodies (arrows) are readily observed on the surface of RTD MNs, while they are absent from Ctrl MNs at two different magnifications (lower magnification in column **A**, higher magnification in column **B**). Scale bar, 2 μm.

To study cell death process in the cell type mostly affected by RTD, we differentiated iPSCs into MNs and examined them by SEM. Patient-derived MNs showed dramatically altered morphological features, including evaginations or membrane blebbing, and cell shrinkage, hallmarks of apoptotic process (Fig. 3). Moreover, while Ctrl cells appeared firmly adherent to the substrate and formed an intricate network of cell bodies and neurites, RTD cells were often loosely juxtaposed to each other and to the underlying substrate (Matrigel), and extended shorter neurites, consistent with previous studies [8, 11]. Filopodia-like fine structures also emerged from RTD cell bodies.

**Fig. 4:**
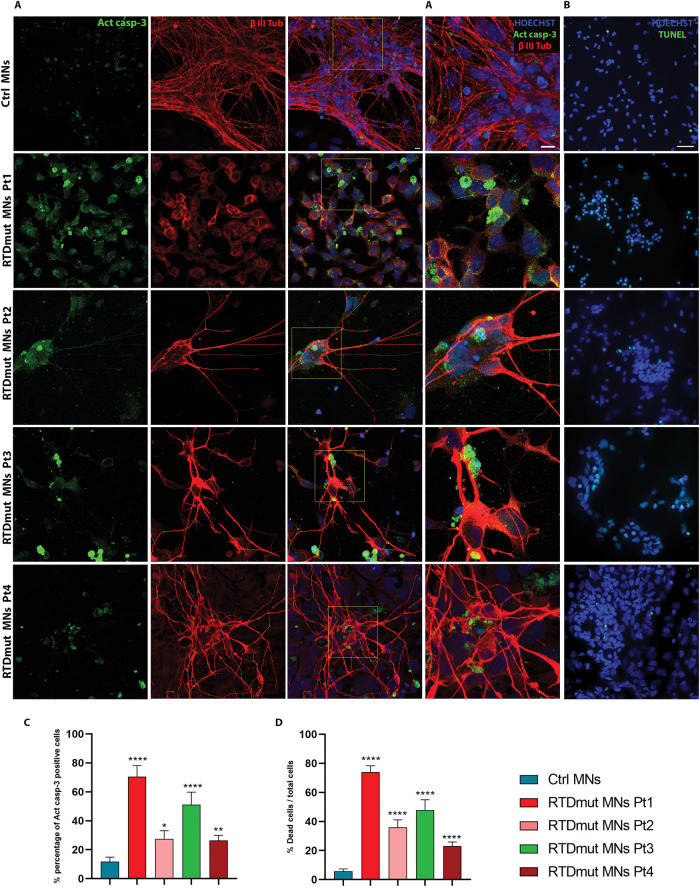
Apoptotic cell death in RTD MNs. **A** Confocal microscopy after double IF, using anti- Act casp-3 (in green) and anti-β III-Tubulin (in red) demonstrates remarkable immunoreactivity to the apoptotic marker in RTD cells. Higher magnification merge images (A’ column), clearly show localization of Act casp-3 in the cytoplasm of RTD cells and in smaller structures, identified as apoptotic bodies. A minimum of *n* = 3 and 9 images were quantized. Scale bar, 10 µm. **B** Confocal images after TUNEL assay show remarkably more numerous positive nuclei (in green) in RTD cells, as compared to healthy cells. All nuclei were labeled in blue by HOECHST. A minimum of 11 images and 2200 cells were counted. Scale bar, 50 µm. **C**, **D** Bar graphs showing the percentage of Act casp-3 and TUNEL positive cells, respectively. Data are presented as the mean ± SEM of three experiments (*n* = 3) and analyzed by Mann–Whitney test. ****p ≤ 0.0001; ***p ≤ 0.001; **p ≤ 0.01; *p ≤ 0.05.

To characterize the cell death process in RTD MNs, we performed Act casp-3 IF (Fig. 4A) and TUNEL assays (Fig. 4B) on healthy and RTD MNs. Confocal images showed significantly increased percentage of Act casp-3 positive cells in RTD MNs, as compared to controls (Fig. 4C). Double IF, using anti-β III Tub also showed a decreased signal in MNs derived from Pt1 iPSCs. Apoptotic bodies and nuclei fragmentation were readily detected in MNs from all RTD patients (Fig. 4A’). Consistently, TUNEL assays revealed the presence of a remarkably increased number of apoptotic nuclei in RTD MN, as compared to Ctrl cells (Fig. 4B, D).

**Fig. 5:**
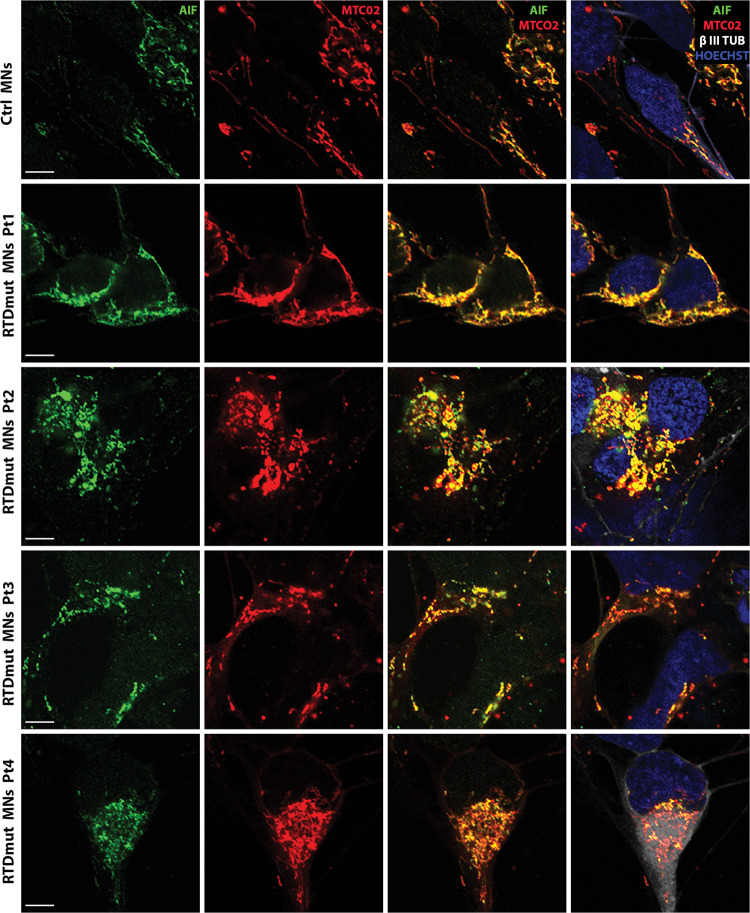
Confocal images of immunofluorescence analysis of AIF (green), MTC02 (red) and β III TUB (white) localization. Hoechst (blue) was used to counterstain nuclei. Images show almost total overlap of AIF and MTCO2 signals (best seen in the third column). Scale bar, 5 µm.

All the described alterations related to the activation of the apoptotic pathway, were consistently more severe in Pt1 MNs, though statistically significant in all RTD differentiated cells. To ascertain caspase involvement in apoptotic cell death in RTD, thereby excluding caspase-independent pathways, we addressed possible implication of AIF, based on the notion that this molecule is also supposed to be released by dysfunctional mitochondria. Specifically, IF labeling was used to assess changes in AIF expression and localization in RTD MNs. Triple IF, using anti-AIF, anti-mitochondria antibody (MTC02) and anti-βIII Tubulin, showed overall co-localization of AIF/MTCO2 in RTD, as well as in Ctrl MNs (Fig. 5). Interestingly, little or no nuclear AIF localization was detected in any cell.

**Fig. 6:**
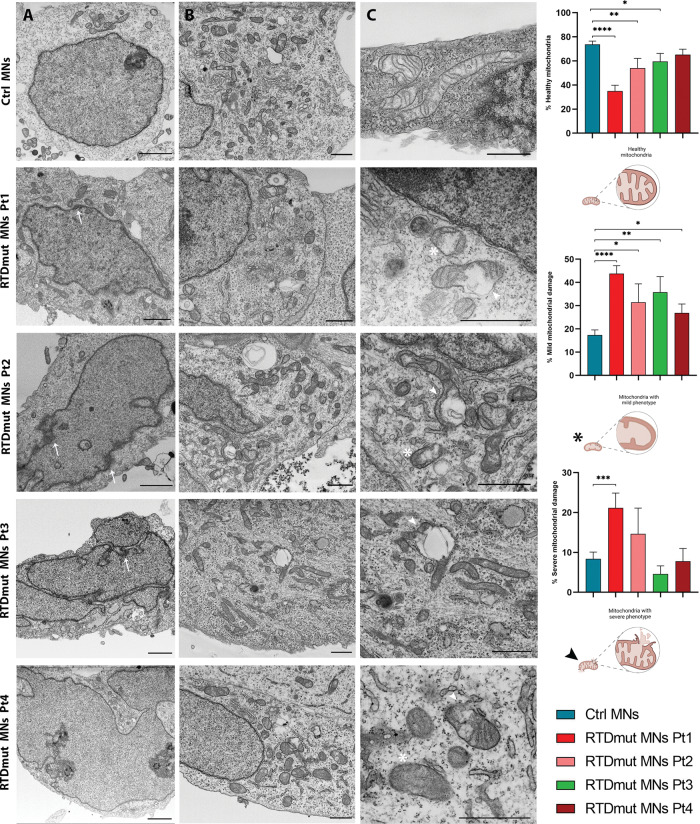
FIB/SEM micrographs show abnormal ultrastructural features of RTD MNs. (**A** column) Electron micrographs show abnormal nuclear morphology in RTD MNs, characterized by an irregular shape, often lobed. (**A** column, arrow) The presence of chromatin clumps is reminiscent of apoptotic process. (**B**, **C** columns) Ctrl MNs show healthy mitochondria with well-developed *cristae* while in RTD MNs several damaged organelles with either dilated (asterisks) or disrupted (arrowheads) *cristae* are observed. Patients’ MNs show a significantly higher number of damaged mitochondria with severe or mild phenotype, compared to Ctrl cells that have a higher percentage of healthy organelles. The percentages of mitochondria with mild or severe phenotype were obtained by manually counting and classifying mitochondria of Ctrl and RTD MNs. Data are presented as the mean ± SEM and analyzed by *T* tests. ****p ≤ 0.0001; ***p ≤ 0.001; **p ≤ 0.01; *p ≤ 0.05. **A** Scale bars, 2 µm; (**B**, **C**) Scale bars, 1 µm.

The series of irreversible events triggered by activation of the apoptotic pathway include chromatin condensation accompanied by DNA degradation. FIB/SEM analyses allowed us to study the nuclear and cytoplasmic fine morphology of Ctrl and RTD MNs (Fig. 6). The latter were characterized by lobed nuclei, surrounded by an indented nuclear envelope. Such ultrastructural features remarkably differed from that observed in Ctrl cells, which instead showed rounded-shape nuclei and a regularly arranged nuclear envelope. Furthermore, intranuclear inhomogeneities were observed, possibly corresponding to chromatin fragmentation (Fig. 6A, arrow). Ultrastructural analysis of RTD MNs also confirmed mitochondrial abnormalities associated with RTD phenotype. Patients’ cells contained few mitochondria, often immature or degenerated, with the presence of few, fragmented or swollen *cristae*, and with an altered organization of the inner mitochondrial membrane (Fig. 6B, C). To make a semi-quantitative evaluation of RTD-associated phenotype severity, we classified mitochondrial morphology as healthy, mildly affected and disrupted, based on the following features: *cristae* fragmentation and outer membrane ruptures. Statistical analyses confirmed the presence of a significantly greater number of either severely or mildly damaged organelles in RTD compared to Ctrl cells.

**Fig. 7:**
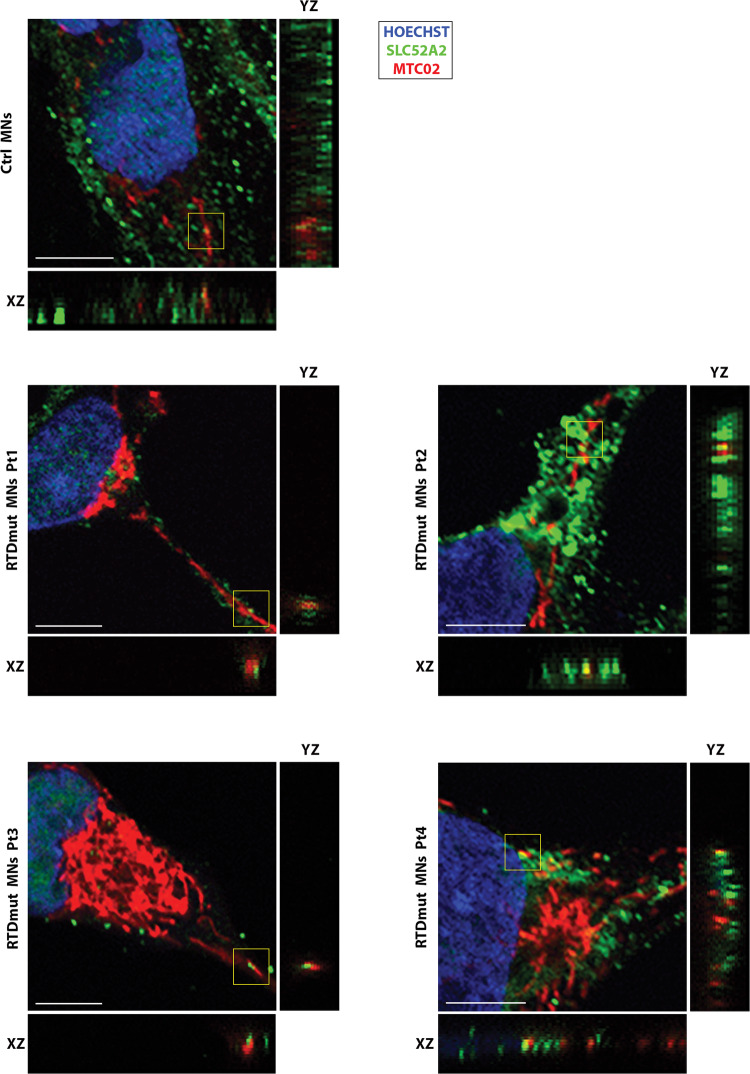
Confocal analysis of RFVT3 and mitochondrial distribution in MNs. Double IF against MTCO2 (red) and SLC52A2 (green) show partial co-localization, as shown in XYZ stacks (XZ and YZ). Hoechst (blue) was used to counterstain nuclei. Scale bar, 5 µm.

Considering the mitochondrial damage observed in RTD MNs carrying *SLC52A2* mutations, resulting in defective RFVT2 transporter, we hypothesized that the morphological alterations could be related to the presence and function of RFVT2 protein in these organelles. To test this hypothesis, we performed double IF using anti-RFVT2 antibody and anti- MTC02 antibody. Interestingly, RFVT2 displayed a granular immune distribution, which in cells derived from RTD Pt2 tends to form clusters with abnormal distribution. Moreover, the signal is partially co-localizing with the mitochondrial marker in control and patient-derived neurons. Representative confocal images are shown in Fig. 7.

## Discussion

Neurodegenerative diseases are characterized by the progressive dysfunction and loss of specific neuronal populations, caused by various pathogenic factors. Irrespective of the etiology, the predominant mechanism of cell death is apoptosis, which is generally triggered by endogenous stimuli [24]. Among intrinsic pathways, mitochondrial-mediated mechanisms are particularly involved in MN diseases due to the crucial role played by these organelles in cell survival and energy metabolism, particularly in MNs [25]. This class of disorders includes RTD, which is characterized by biallelic mutations of the riboflavin transporter RFVT2 or RFVT3 [1].

In the present work, we addressed possible activation of the apoptotic process in RTD cells. Based on previous studies on iPSCs obtained from RTD patient-derived fibroblasts, showing hallmarks of activated apoptotic process [9], we further investigated morpho-functional and ultrastructural features of RTD cells, focusing on the cell compartments mostly involved in apoptotic processes, as nucleus, mitochondria, and cell surface. In either RTD iPSCs and RTD MNs, FIB/SEM micrographs showed plasma membrane blebs, known to be associated with apoptotic bodies [23]. These results are consistent with previous data documenting cell-cell contact defects, loss of ZO-1 mediated tight junctions and abundant extracellular vesicles in RTD iPSCs [9]. Apoptotic activation was assessed by TUNEL assay, which detects double-stranded DNA fragments that are generated by endonucleases in dying cells [26]. We detected an increased percentage of TUNEL positive cells in RTD iPSCs and MNs, compared to their healthy counterparts, confirming an activation of the programmed cell death pathway. Consistently, FIB/SEM ultrastructural investigation provided detailed information related to nuclear alterations. Unlike control cells, RTD MNs frequently showed lobed nuclei, containing chromatin clumps, similar to those widely described for apoptotic cells [27].

Activation of apoptosis may well correlate with impaired transport of RF, as the precursor of FAD and FMN, which play crucial roles in several pathways, including redox reactions and NAD synthesis. In the very first work performed on RTD cells, Rizzo and Coll [28]. analysed the specific activities of individual respiratory-chain complexes in RTD MNs and showed decreased activity of cytochrome c oxidase (COX, complex IV). Even though such alteration is apparently uncorrelated to Electron Chain Transport (ETC) defects [28], it may contribute to reduced energy generation, in analogy with other neurodegenerative diseases, particularly Alzheimer’s disease [29, 30]. The altered process likely leads to overproduction of reactive oxygen species (ROS), not counter-balanced by efficient antioxidant response [10]. Indeed, we previously addressed RTD-associated redox status, demonstrating abnormally high superoxide anion concentration in RTD cells, as assessed by MitoSOX Red probe [9]. Furthermore, we showed that RF deficiency also interferes with the maintenance of reduced glutathione (GSH), a major natural ROS-scavenging molecule. Specifically, we detected significantly lower expression levels and abnormal immunofluorescence distribution of GSH in RTD vs. Ctrl cells [9]. Such redox imbalance in patient cells is accompanied by abnormal mitochondrial membrane polarization state, evaluated by JC-1 experiments [9]. Altogether, these findings strongly argue for apoptosis triggering, providing a sound basis for our data, showing activation of this death pathway.

In addition to this, we performed electron microscopic analyses to investigate the fine structure of MNs from RTD patients. Consistent with data collected on iPSCs and MNs showing mitochondrial involvement in RTD pathogenesis [9, 10], dramatic mitochondrial swelling associated with *cristae* derangement was observed in RTD cells. Disrupted mitochondrial morphology is totally consistent with a caspase-dependent mechanism, triggered by Cyt *c* release from the intermembrane space. Consistent with this hypothesis, Act casp-3 immunolocalization resulted in remarkably stronger fluorescent signal in RTD iPSCs and MNs, as compared to Ctrl cells. Since MOMP may also cause leakage of AIF, mediating caspase-independent mechanisms [31], we explored its distribution in RTD and Ctrl cells. However, this protein appeared to be sequestered in mitochondrial compartment, as assessed by double IF with the MTCO marker, suggesting that AIF contribution as an endonuclease to cell death is not relevant to RTD. To relate overall cell sufferance involving mitochondrial damage with pathological *SLC52A2* mutations carried by the patients, we studied the intracellular IF localization of the transporter RFVT2, with special reference to mitochondria. Confocal analyses showed wide intracellular distribution of RFVT2, which was characterized by a granular appearance in the soma and along the neurites, supporting its role in intracellular transport of RF, possibly through membrane vesicular trafficking. Intriguingly, areas of partial colocalization of RFVT2 with MTC02 were present in Ctrl and RTD cells, suggesting a role for the transporter in carrying flavins into mitochondria. In addition, relatively large RFVT2 aggregates were found in Pt.2 MNs, prompting further work to investigate such peculiarly increased RFVT2 signal.

**Fig. 8:**
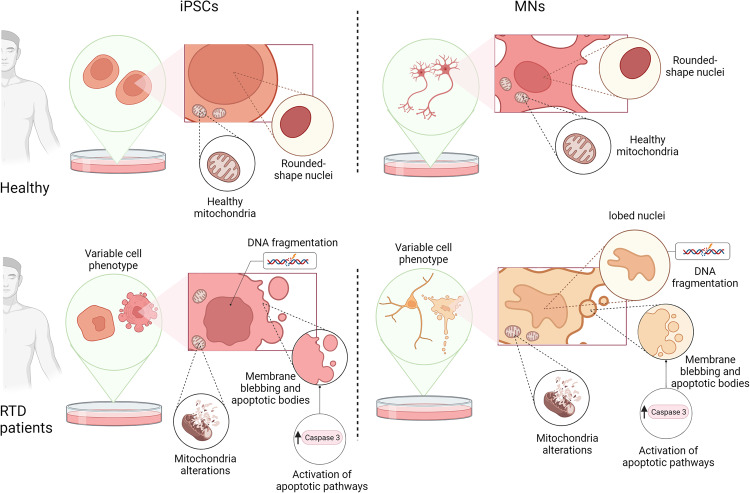
Schematic drawing showing the morphological changes and the altered metabolic pathways in RTD cells. The figure was created with BioRender.com.

Taken together, our findings support a model (Fig. 8), in which RTD results in an endophenotype – measurable “*trait d’union*” between the genotype and phenotype – which is characterized by cellular features strongly arguing for massive apoptotic process. Surface alterations, abnormal nuclear morphology, DNA fragmentation and mitochondrial damage are among the most remarkable changes indicating programmed cell death. While further studies are needed to clarify additional aspects of neurodegeneration and to understand the prevalent pathogenic mechanism, we encourage addressing the role of pro-survival pathways in RTD cells, e.g., the autophagic process, with special reference to mitophagy.

## Materials and methods

### Maintenance of iPSCs

For this study, we use iPSCs derived from skin biopsies of four RTD patients as described in Magliocca et al., (submitted). The patients carry the following mutations: RTDmut Pt1 (c.155 C > T and c.1255 G > A; SLC52A2mut p. S52F; p. G419S); RTDmut Pt2 (c.155 C > T and c.935 T > C; SLC52A2mut p. S52F; p. L312P); RTDmut Pt3 (c.505 C > T and c.1030_1031del; SLC52A2mut p. Arg169Cys; p. Leu344AlafsX100); RTDmut Pt4 (c.505 C > T and c.593 G > A; SLC52A2mut p. Arg169Cys; p. Trp198Ter). As a reprogramming method, the non-integrative episomal technology (#SC301A-1, Minicircle DNA and mc-iPS Cells, Euroclone, Milan, Italy) was used. The iPSCs were plated on 6-well Matrigel-coated multiwells (BD Biosciences, San Jose, USA), kept in culture in mTeSR1 plus Basal Medium (#05826, Stem Cell Technologies, Vancouver, Canada) in an incubator at 37 °C with 5% CO_2_ and 21% O_2_. When iPSCs reached about 70% confluence, they were dissociated using EDTA (ethylenediaminetetraacetic acid) and plated on newly Matrigel-coated wells.

### Differentiation of iPSCs into Motor Neurons

iPSCs were differentiated into MNs by adapting the protocol proposed by Corti et al. [32]. Cells were grown for 10 days in NeuroCult NS-A Basal Human Medium (#05750, Stem Cell Technologies), after which retinoic acid (#R2625, Sigma Aldrich, St. Louis, MI, USA) was added. The culture medium was replaced on alternate days until day 17 when, in addition to 0.1 µΜ retinoic acid, 2 µΜ dorsomorphin (#P5499, Sigma Aldrich) and 3 ng/ml activin A (#120-14E, PeproTech, Rocky Hill, CT, USA) were added. Starting from day 25, until the end of the differentiation protocol, the culture medium was replaced with BrainPhys Neuronal Medium (#05790, Stem Cell Technologies), containing 200 μM ascorbic acid (#A4403, Sigma Aldrich), 2 μg/ml GDNF (#450-10, PeproTech), 10 ng/ml BDNF (#450-02, PeproTech), SM1 (#05711, Stem Cell Technologies) and N2 (#17502-001, Thermofischer Scientific, Waltham, MA, USA).

### TUNEL assay

For TUNEL assays (#G3250, Promega, Madison, WI, USA), cells were plated on slides and fixed with 4% formaldehyde solution in phosphate buffer saline (PBS) for 10 min, at room temperature (RT). Cells were permeabilized by 0.1% Triton X-100 for 10 min at 4 °C, then washed with PBS. Equilibration Buffer was added to samples for 5 min at RT, prior to incubation with 45 µl Equilibration Buffer, containing 5 µl Nucleotide Mix and 1 µl TdT, for 1 h at 37 °C. The reaction was stopped by adding 2X SSC for 15 min and nuclei were contrasted by Hoechst (1: 10000 for 10 min).

### Immunofluorescence and confocal microscopy

After differentiation, RTD and healthy iPSCs and MNs were fixed with 4% formaldehyde for 10 min at RT, then incubated with a blocking and permeabilizing solution, composed of 5% Bovine Serum Albumin (BSA, #10775835001, Roche, Basilea, Switzerland); 0.1% Triton X-100 (Sigma-Aldrich) in PBS, for 1 h at RT. Cells were treated with the primary antibody of interest, diluted in PBS containing 3% BSA, as follows: 1:100 Activated Caspase 3 #C8487, Sigma Aldrich; 1:100 AIF #4642, Cell Signaling (Danvers, MA, USA); 1:500 βIII TUB #T8578, Sigma Aldrich; 1:200 Mitochondria #NBP2-32982, Novus BIO (Centennial, CO, USA), 1:100 SLC52A2 #CSB PA060150, CusaBio (Houston, TX, USA). Slides were immersed in buffer, then incubated with appropriate secondary antibodies, conjugated to either of the following, diluted 1:500 in PBS, for 1 h: Alexa Fluor 488, Alexa Fluor 555 or Alexa Fluor 647 (Invitrogen, Carlsbad, CA, USA). Nuclei were counterstained using 1:10000 Hoechst (#33342, Invitrogen) in PBS for 10 min, at RT. After mounting with 1:1 PBS/Glycerol, slides were observed in a Leica TCS SP5 (Leica Microsystems, Wetzlar, Germany) confocal microscope or Olympus FV3000 (Evident Europe GmbH, Olympus, Microsystems, Hamburg, Germany) equipped with 405 nm-488 nm-561 nm and 640 nm diode lasers. Representative images were captured and assembled using Adobe Photoshop CS6 software (Adobe Systems Inc., San Jose, CA, USA).

### Western blot analyses

For western analysis, cells were lysed in buffer composed by RIPA (#S-R0278, Sigma) protease inhibitor cocktail (Roche, Basilea, Swiss) and 0.5 mM Sodium Orthovanadate. cell extracts were separated by 10% sodium dodecyl sulfate–polyacrylamide gel electrophoresis and transferred to nitrocellulose membranes (#1704159, Bio-Rad, Hercules, CA). Membranes were blocked in 5% milk for 1 h at RT and incubated with primary antibodies overnight at 4 °C. Blots were incubated with appropriate secondary antibodies (#111-035-003, Jackson ImmunoResearch, United Kingdom) for 1 h at RT and stained with SuperSignal West Pico Chemiluminescent Substrate (Pierce Biotechnologies, Massachusetts, USA). The following primary antibodies were used: Act casp-31:1000 overnight (#C8487, Sigma Aldrich) and GAPDH 1:10000 overnight (#ab8245, Abcam).

### Electron microscopy

For SEM analysis, cells were plated on coverslips, fixed in 2.5% glutaraldehyde in 0.1 M cacodylate buffer, pH 7.4, for 45 min at 4 °C. After washing, cells were post-fixed with 1% OsO_4_ in the same buffer for 45 min, at 4 °C in the dark, then dehydrated by ethanol and hexamethyldisilazane (HDMSO). Air-dried slides were mounted on metal stubs by bi-adhesive carbon discs and gold-coated by Emitech K550 Sputter Coater. Electron micrographs were acquired by a Gemini 300 SEM (Carl Zeiss AG, Jena, Germany), detecting secondary electrons with an operating voltage of 5 kV.

For focused ion beam/scanning electron microscopic (FIB/SEM) analyses, cells were plated in chamber slides (Lab-Tek™ II Chamber Slide System, ThermoFischer) and fixed in a mixture of 2% formaldehyde, 0.5% glutaraldehyde in 0.1 M cacodylate, pH 7.4, for 45 min, at 4 °C. After washing and post-fixation performed as above, samples were contrasted with UranyLess (Electron Microscopy Science, Foster City, CA, USA), for 1 h at 4 °C. Cells were dehydrated in ethanol, and infiltrated by a mixture 1:1 of ethanol and epoxy resin (Sigma-Aldrich, Cat# 45359-1EA-F, Burlington, MA, USA), for 1 h at RT, then embedded in absolute resin. After polymerization at 60 °C for three days, samples were selectively milled using FIB column, operated at a voltage of 30 kV and a current of 9.3 nA, to expose regions of interest to be imaged. Micrographs were acquired by SEM column, detecting backscattered electrons at a working distance of 2 mm and using the Everhart-Thornley detector (ETD) with a voltage of 2 kV and a current of 0.34 nA. Images were assembled using Adobe Photoshop CS6 software (Adobe Systems Inc., San Jose, CA, USA).

### Statistical analyses

LAS X software was used to acquire and quantify fluorescence images, while for immunoblotting analysis, Image J software was used to quantify band staining. Statistical analyses were performed using Prism software (GraphPad Software, Inc., La Jolla, CA, USA), followed by parametric (Student’s *t* test, one-way ANOVA) or non-parametric (Mann–Whitney, Kruskal-Wallis) tests, to compare sample groups. For FIB/SEM analyses, approximately 20 cells per sample were analyzed. For IF, TUNEL assay and WB, a minimum of 3 technical and 3 biological replicates were performed for all experiments. Data were expressed as mean and standard error of the mean ± SEM of n ≥ 3 independent experiments and defined as ****p ≤ 0.0001; ***p ≤ 0.001; **p ≤ 0.01; *p ≤ 0.05.

## Data Availability

The datasets generated during and/or analysed during the current study are available from the corresponding author on reasonable request.
